# The impact of COVID-19 on medical students’ practical skills and hygiene behavior regarding venipuncture: a case control study

**DOI:** 10.1186/s12909-022-03601-6

**Published:** 2022-07-19

**Authors:** Annika Meyer, Christoph Stosch, Andreas R. Klatt, Thomas Streichert

**Affiliations:** 1 Faculty of medicine and university hospital, department of clinical chemistry, University of Cologne, Kerpener Str. 62, 50937 Cologne, Germany; 2 Faculty of medicine and university hospital, Interprofessional Skills Lab and Simulation center (KISS), University of Cologne, Joseph-Stelzmann-Straße 9a, 50931 Cologne, Germany

**Keywords:** Medical student, COVID-19, Hygiene, Practical skills, Objective structured clinical examination (OSCE), Online teaching, Venipuncture

## Abstract

**Background:**

Despite their importance to current and future patient care, medical students’ hygiene behaviors and acquisition of practical skills have rarely been studied in previous observational study. Thus, the aim of this study was to investigate the potential impact of the COVID-19 pandemic on medical student’s hygiene and practical skills.

**Methods:**

This case-control study assessed the effect of the COVID-19 pandemic on hygiene behavior by contrasting the practical skills and hygiene adherence of 371 medical students post the pandemic associated lockdown in March 2020 with that of 355 medical students prior to the SARS-CoV-2 outbreak. Students’ skills were assessed using an objective structured clinical examination (OSCE). Their skills were then compared based on their results in hygienic venipuncture and the total OSCE score.

**Results:**

During the SARS-CoV-2 pandemic, medical students demonstrated an increased level of compliance regarding hand hygiene before (prior COVID-19: 83.7%; during COVID-19: 94.9%; *p* < 0.001) and after patient contact (prior COVID-19: 19.4%; during COVID-19: 57.2%; *p* = 0.000) as well as disinfecting the puncture site correctly (prior COVID-19: 83.4%; during COVID-19: 92.7%; *p* < 0.001). Prior to the pandemic, students were more proficient in practical skills, such as initial venipuncture (prior COVID-19: 47.6%; during COVID-19: 38%; *p* < 0.041), patient communication (prior COVID-19: 85.9%; during COVID-19: 74.1%; *p* < 0.001) and structuring their work process (prior COVID-19: 74.4%; during COVID-19: 67.4%; *p* < 0.024).

**Conclusion:**

Overall, the COVID-19 pandemic sensitized medical students’ attention and adherence to hygiene requirements, while simultaneously reducing the amount of practice opportunities, thus negatively affecting their practical skills. The latter development may have to be addressed by providing additional practice opportunities for students as soon as the pandemic situation allows.

**Supplementary Information:**

The online version contains supplementary material available at 10.1186/s12909-022-03601-6.

## Highlights

- The COVID-19 pandemic sensitizes medical students to hygienic aspects of venipuncture.

- The hygiene compliance of medical students is proportional to the duration of the COVID-19 pandemic.

- During the COIVD-19 pandemic, medical students performed worse in non-hygienic tasks.

**Supplementary Information:**

The online version contains supplementary material available at 10.1186/s12909-022-03601-6.

## Background

Over the last century, the world population has repeatedly been confronted with pandemics primarily targeting the respiratory system, such as Influenza A, SARS, MERS-CoV as well as the current COVID-19 pandemic. As these pandemics are associated with high mortality, reducing the transmission of the virus is crucial [1].

“Social distancing” and the implementation of hygiene standards have proven to be reliable precautionary measures to reduce the spread of SARS-CoV-2 [2]. Hence, lockdowns have been implemented all over the world [3–5]. Thus, SARS-CoV-2 has largely impacted and restricted public life on a global scale and made previously contact-based medical teaching of basic practical skills such as venipuncture infeasible [6, 7].

Due to the nature of their work, healthcare professionals are at a particularly high risk of exposure to SARS-CoV-2, as well as susceptible to increased stress levels which result from a pandemic related increased workload [8, 9]. To alleviate the strain on healthcare workers, medical students have reportedly volunteered to assist them during this health care crisis [10–13]. Since proper hygiene, or lack thereof, plays a fundamental role in the transmission of pathogens, such as SARS-CoV-2, medical students’ hygiene adherence is highly relevant to pandemic containment [14]. While previous pandemics have had little impact on medical students, the literature suggests that SARS-CoV-2 is now affecting medical students’ hygiene awareness, knowledge and compliance to a greater extent [8, 15–22]. Nonetheless, these study results are based on surveys and not on other study models, such as observational studies. Moreover, European medical students have not been surveyed so far [15, 19, 23–27]. Therefore, European medical students’ adherence to hygiene protocols during the COVID-19 pandemic seems insufficiently investigated.

## Methods

**Fig. 1:**
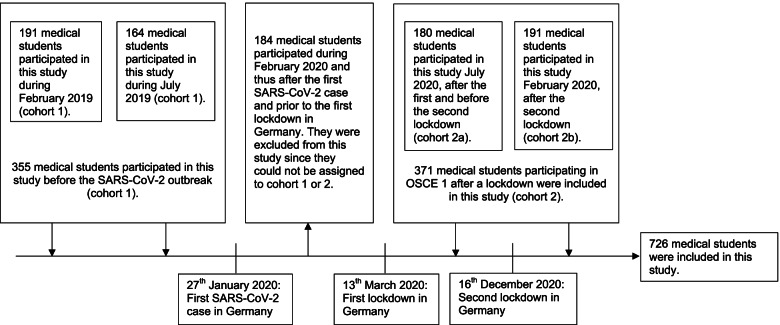
Study cohort in relation to temporal SARS-CoV-2 progression in Germany, 2019–2021 (*N* = 726)

This unicentric case-control study examined the venipuncture skills and level of hygiene-compliance of third year medical students who participated in OSCE at the University of Cologne before and during the COVID-19 pandemic. The data was collected during OSCE, which is a practical test consisting of seven five-minute test stations and usually takes place in the third year of medical school in Cologne. The examined practical skills were assessed by medical students during their practical year. Failure to pass this exam was of no academic relevance to the tested students. Students are prepared for this test in a practical course, which is designed to teach skills such as hygienic venipuncture and hand disinfection. In total 910 medical students underwent OSCE from February 5th, 2019, to February 19th, 2021, in Cologne. 184 medical students participating in the OSCE in February of 2020 were excluded from this study, since they were assessed after the first SARS-CoV-2 case and before the implementation of pandemic containment measures in Germany. Therefore, the 184 medical students could not be assigned to either the control group or the lockdown group. In conclusion, only the data of 726 medical students were included in this study. Medical students who participated in OSCE prior to the first diagnosed SARS-CoV-2 case in Germany provided the control group (cohort 1). On contrary medical students who took part in OSCE after the first lockdown in March 2020 provided the investigated cohort (cohort 2). A subgroup analysis of cohort 2 allowed the comparison of medical students’ hygiene behavior at different stages of the pandemic (cohort 2a: after first lockdown; cohort 2b: after second lockdown) (Fig. 1).

All data were collected using the same standardized questionnaire (Additional file 1: Appendix 1). The focus of this study was hygienic venipuncture as part of indwelling venous cannulation or blood extraction and the total OSCE score.

Based the results of the Kolmogorov-Smirnov-test, all data were not normally distributed (Additional file 2: Appendix 2). Accordingly, differences between the control and the investigated group were assessed using Mann-Whitney-U test. *P*-values < 0.05 were considered statistically significant. Frequencies and percentages were demonstrated by categorical parameters, while continuous variables were expressed by their mean and standard deviation.

For all statistical analyses, IBM SPSS Statistics version 27 (SPSS Inc., Chicago, Illinois) was used.

All medical students partaking in this study were of legal age. Neither the students nor the examiners knew the purpose of this study prior to the data collection. The local ethics committee reviewed and approved this research project on August 20, 2021, prior to its initiation (approval number: 21–1332). Upon enrollment in the medical program at the University of Cologne, students consented in writing to data collection and analysis. The examined retrospective data were analyzed pseudonymously.

The aim of this study was to compare medical students’ compliance with standard hygiene protocol regarding venipuncture and other hygienic tasks before and during the COVID-19 pandemic using their results in OSCE.

## Results

Out of 726 medical students partaking in OSCE, 355 (48.9%) participated before (cohort 1) and 371 (51.1%) after the first lockdown in Germany during March 2020 (cohort 2). The relative score of both cohorts showed only a slight deviance regarding their relative score for OSCE (pre-lockdown: 62.53 ± 8.47; post-lockdown: 67.13 ± 7.22; *p* < 0.001) and venipuncture (pre-lockdown: 71.87 ± 15.85; post-lockdown: 74.36 ± 17.4; *p* < 0.001).

After the lockdown, 352/371 medical students (94.9%) disinfected their hands before patient contact and 212/371 (57.2%) after patient contact, whereas prior to the lockdown only 297/355 students (83.7%) did so before and 69/355 (19.4%) after patient contact (*p*-value for hand disinfection prior to patient contact < 0.001; *p*-value for hand disinfection after patient contact = 0.000).

After lockdown, significantly more students disinfected their hands prior patient contact (94.95%) compared to after patient contact (57.2%) (*p* < 0.001). Moreover, the number of students disinfecting the puncture site increased post lockdown. (pre-lockdown: 296/355, 83.4%; post-lockdown: 344/371, 92.7%; *p* < 0.001). No significant difference was found between the groups regarding the observance of the 30-second exposure time of the disinfectant. Here, both demonstrated a high level of hygiene compliance (pre-lockdown: 94.9%; post-lockdown: 97.6%; *p* < 0.06).

There was no statistically significant difference between the two cohorts regarding the preparation of the materials (pre-lockdown: 176/355, 49.6%; post-lockdown: 201/371, 54.2%; *p* = 0.691), the use of sterile and unbent puncture needles (pre-lockdown: 303/355, 85.4%; post-lockdown: 311/371, 83.9%; *p* = 0.57) as well as discarding the puncture needle (pre-lockdown: 126/355, 35.5%; post-lockdown: 149/371, 40.2%; *p* = 0.177).

**Fig. 2:**
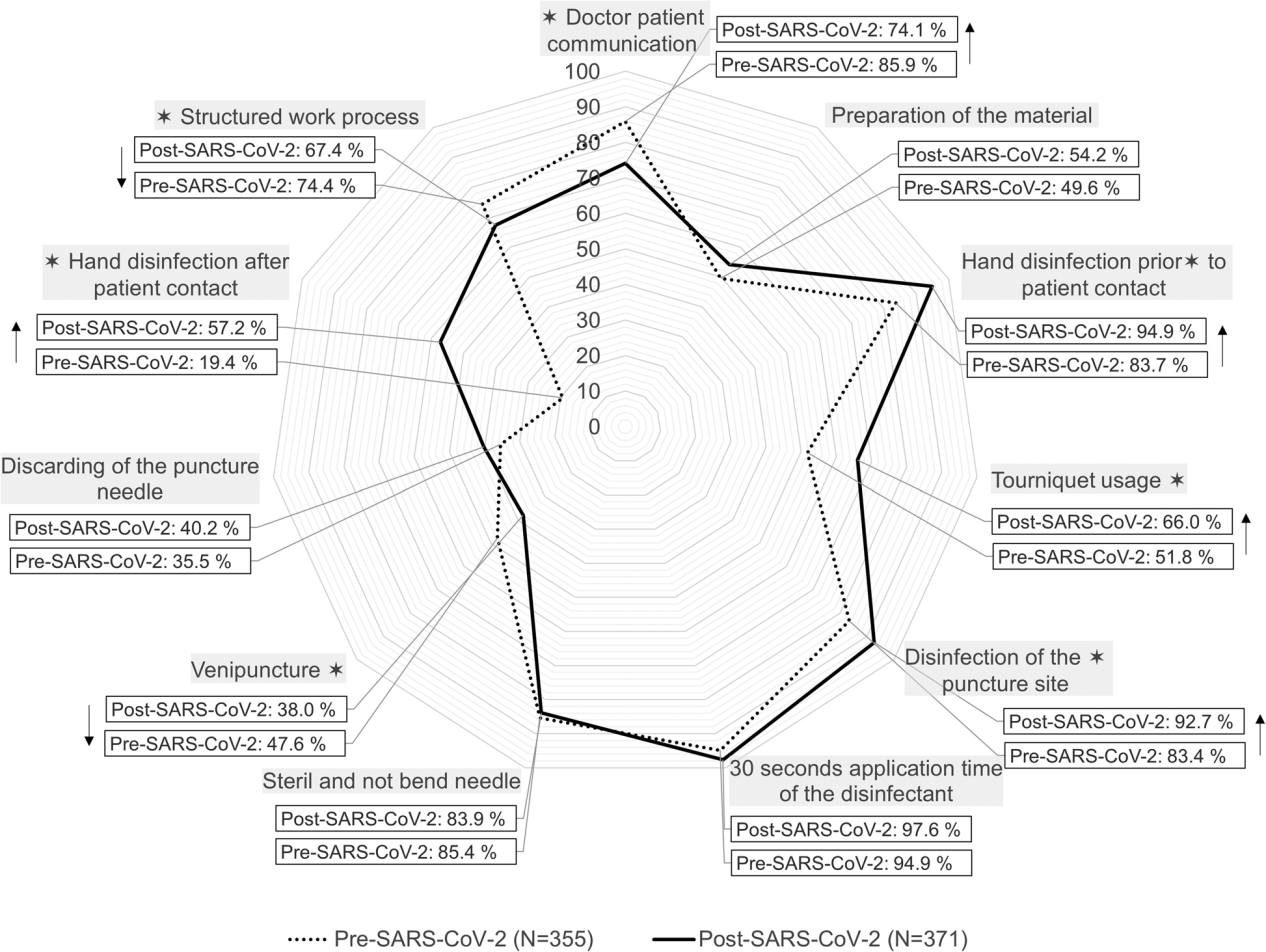
Frequency of correct performance before the COVID-19 pandemic and post-lockdown in Germany, 2019–2021 (*n* = 726). The dotted line represents the number of medical students performing a skill correctly before the COVID-19 pandemic (Pre-SARS-CoV-2). The solid line represents the number of medical students performing a skill correctly after the first lockdown (Post-SARS-CoV-2). Frequencies are shown in percentages. Statistically significance determined by the chi-square test is marked with *. Upward arrows indicate improvement in medical students’ skills after the SARS-CoV-2 outbreak, while downward arrows indicate better results prior the pandemic

Notably, successful venipuncture (pre-lockdown: 169/355, 47.6%; post-lockdown: 141/371, 38%; *p* = 0.041), doctor-patient communication (pre-lockdown: 305/355, 85.9%; post-lockdown: 275/371, 74.1%; *p* < 0.001) and structure in the work processes (pre-lockdown: 264/355, 74.4%; post-lockdown: 250/371, 67.4%; *p* = 0.024) were less frequently demonstrated by the students partaking after the first lockdown. Thus, applying a tourniquet was the only practical skill that medical students were more proficient at after the first lockdown than before (pre-lockdown: 184/355, 51.8%; post-lockdown: 245/371, 66%; *p*-value < 0.001) (Table 1, Fig. 2).

**Table 1:** Hygienic venipuncture of medical students pre- and post-SARS-CoV-2 in Germany during 2019–2021 (*N* = 726)

	All	Before SARS-CoV-2	After SARS-CoV-2	*P*-value
Number of participants	(N = 726)	(*N* = 355)	(*N* = 371)	
Winter semester	382 (52.6)	191 (53.8)	191 (48.5)	
Summer semester	344 (47.4)	164 (46.2)	180 (51.5)	
Scores for other Stations				
Mean relative OSCE score	64.88 ± 8.18	62.53 ± 8.47	67.13 ± 7.22	< .001
Mean relative score in venipuncture	72.25 ± 16.79	71.87 ± 15.85	74.36 ± 17.4	< .001
Doctor-patient communication				
The patient is not informed about the procedure	146 (20.1)	50 (14.1)	96 (25.9)	< .001
The patient is informed about the procedure	580 (79.9)	305 (85.9)	275 (74.1)	
Preparation of the material				
More than one material or the sharp-safe is missing	211 (29.1)	85 (23.9)	126 (34)	.691
At least one material is missing	138 (19)	94 (26.5)	44 (11.9)	
Complete and correct preparation of the material	377 (51.9)	176 (49.6)	201 (54.2)	
Hygienic hand disinfection and medical gloves prior to patient contact				
Neither hygienic hand disinfection nor medical gloves	16 (2.2)	13 (3.7)	3 (0.8)	< .001
Medical gloves without prior hand disinfection	61 (8.4)	45 (12.7)	16 (4.3)	
Hand disinfection and medical gloves	649 (89.4)	297 (83.7)	352 (94.9)	
Tourniquet usage				
The tourniquet is not applied or disposed of correctly	67 (9.2)	51 (14.4)	16 (4.3)	< .001
The tourniquet is not applied, while the needle is pulled before disposing of the tourniquet	230 (31.7)	120 (33.8)	110 (29.6)	
The tourniquet is applied and disposed in the correct manner	429 (59.1)	184 (51.8)	245 (66)	
Disinfection of the puncture site				
The puncture site is not disinfected, or it is palpated after the disinfection and prior to the venipuncture	86 (11.8)	59 (16.6)	27 (7.3)	< .001
The puncture site is correctly disinfected	640 (88.1)	296 (83.4)	344 (92.7)	
Exposure time of the disinfectant				
30 seconds exposure time is not considered	27 (3.7)	18 (5.1)	9 (2.4)	.06
30 seconds exposure time is considered	699 (96.3)	337 (94.9)	362 (97.6)	
Hygienic needle				
The needle is not sterile or is curved	112 (15.4)	52 (14.6)	60 (16.2)	.57
The needle is sterile and not curved	614 (84.5)	303 (85.4)	311 (83.9)	
Correct venipuncture				
The vein is not punctured	171 (23.6)	81 (22.8)	90 (24.3)	.041
The vein is punctured the second time	245 (33.7)	105 (29.6)	140 (37.7)	
The vein is punctured the first time	310 (42.7)	169 (47.6)	141 (38)	
Discarding the puncture needle				
The needle is not discarded correctly	339 (46.7)	174 (49)	165 (44.5)	.177
The needle is discarded immediately but not correctly	112 (15.4)	55 (15.5)	57 (15.4)	
The needle is discarded immediately and correctly	175 (37.9)	126 (35.5)	149 (40.2)	
Hand disinfection after discarding the medical gloves				
Hands are not disinfected after discarding the medical gloves	445 (61.3)	286 (80.6)	149 (42.9)	.000
Hands are disinfected after discarding the medical gloves	281 (38.7)	69 (19.4)	212 (57.2)	
Structure in the work process				
The work process is not structured	36 (5)	11 (3.1)	25 (6.7)	.024
The work process is partly structured	176 (24.2)	80 (22.5)	96 (25.9)	
The work process is structured	514 (70.8)	264 (74.4)	250 (67.4)	

Counts are described by their frequency and percentage. Continuous variables are described by their mean and standard deviation

Statistic differences between pre- and post-SARS-CoV-2 was tested using the Mann-Whitney-U test

Out of 371 medical students partaking in OSCE during the pandemic (cohort 2), 180 (48.5%) participated after the first lockdown in March 2020 (cohort 2a) and 191 (51.5%) after the start of the second lockdown in December 2020 (cohort 2b).

Medical students performed slightly better in OSCE after the first compared to the second lockdown (first lockdown: 68.23 ± 6.46; second lockdown: 66.1 ± 7.75; *p* = 0.003), while no significant statistical difference could be found between the relative score in venipuncture after the first (74.86 ± 11.3) compared to the second lockdown (73.89 ± 18.23; *p* = 0.833). No statistical difference was also evident in regards to hand disinfection before patient contact (first lockdown: 93.9%; second lockdown: 95.8%; *p* = 0.412), disinfection of the puncture site (first lockdown: 93.3%; second lockdown: 92.1%; *p* = 0.661), consideration of the 30 second disinfectant exposure time (first lockdown: 96.7%; second lockdown: 98.4%; *p* = 0.271), correct tourniquet usage (first lockdown: 67.8%; second lockdown: 64.4%; *p* = 0.354),the use of sterile and unbent needles (first lockdown: 84.4%; second lockdown: 83.2%; *p* = 0.754),discarding the puncture needle (first lockdown: 40.6%; second lockdown: 39.8%; *p* = 0.256). Although the cohorts did not differ significantly in their communication (first lockdown: 78.3%; second lockdown: 70.2%; *p* = 0.073), the number of medical students informing the patient about the procedure decreased by 8.1% during the second lockdown compared to the first.

In terms of structured work (first lockdown: 76.7%; second lockdown: 58.6%; *p* < 0.001) and successful venipuncture (first lockdown: 48.3%; second lockdown: 28.3%; *p* < 0.001), the cohort who participated in OSCE after the first lockdown in March 2020 performed better than their peers partaking during the second lockdown.

In contrast, an improvement in the disinfection of their hands after patient contact could be observed in the medical students participating after the second lockdown as compared to the first (first lockdown: 47.8%; second lockdown: 66%; *p* < 0.001). Materials needed for venipuncture were also more frequently adequately prepared during the second than during the first lockdown (first lockdown: 44.4%; second lockdown: 63.4%; *p* < 0.001) (Table 2).

**Table 2:** Hygienic venipuncture of medical students after the first and second lockdown in Germany during 2020–2021 (*N* = 371)

	First lockdown	Second lockdown	*P*-value
Number of participants	(*N* = 180)	(*N* = 191)	
Scores for other stations			
Relative OSCE Score	68.23 ± 6.46	66.1 ± 7.75	.003
Relative score venipuncture	74.86 ± 11.3	73.89 ± 18.23	.833
Doctor-patient communication			
The patient is not informed about the procedure	39 (21.7)	57 (29.8)	.073
The patient is informed about the procedure	141 (78.3)	134 (70.2)	
Preparation of the material			
More than one material or the sharp-safe is missing	78 (43.3)	48 (25.1)	< .001
At least one material is missing	22 (12.2)	22 (11.5)	
Complete and correct preparation of the material	80 (44.4)	121 (63.4)	
Hygienic hand disinfection and medical gloves prior to patient contact			
Neither hygienic hand disinfection nor medical gloves	1 (0.6)	2 (1)	.412
Medical gloves without prior hand disinfection	10 (5.6)	6 (3.1)	
Hand disinfection and medical gloves	169 (93.9)	183 (95.8)	
Tourniquet usage			
The tourniquet is not applied or disposed of correctly	4 (2.2)	12 (6.3)	.354
tourniquet is not applied, while the needle is pulled before disposing of the tourniquet	54 (30)	56 (29.3)	
The tourniquet is applied and disposed in the correct manner	122 (67.8)	123 (64.4)	
Disinfection of the puncture site			
The puncture site is not disinfected, or it is palpated after the disinfection and prior to the venipuncture	12 (6.7)	15 (7.9)	.661
The puncture site is correctly disinfected	168 (93.3)	176 (92.1)	
Exposure time of the disinfectant			
30 seconds exposure time is not considered	6 (3.3)	3 (1.6)	.271
30 seconds exposure time is considered	174 (96.7)	188 (98.4)	
Hygienic needle			
The needle is not sterile or is curved	28 (15.6)	32 (16.8)	.754
The needle is sterile and not curved	152 (84.4)	159 (83.2)	
Correct venipuncture			
The vein is not punctured	31 (17.2)	58 (30.9)	< .001
The vein is punctured the second time	62 (34.4)	78 (40.8)	
The vein is punctured the first time	87 (48.3)	54 (28.3)	
Discarding the puncture needle			
The needle is not discarded correctly	71 (39.4)	94 (49.2)	.256
The needle is discarded immediately but not correctly	36 (20)	21 (11)	
The needle is discarded immediately and correctly	73 (40.6)	76 (39.8)	
Hand disinfection after discarding the medical gloves			
Hands are not disinfected after discarding the medical gloves	94 (52.2)	65 (34)	< .001
Hands are disinfected after discarding the medical gloves	86 (47.8)	126 (66)	
Structure in the work process			
The work process is not structured	5 (2.8)	20 (10.5)	< .001
The work process is partly structured	37 (20.6)	59 (30.9)	
The work process is structured	138 (76.7)	112 (58.6)	

Counts are described by their frequency and percentage. Continuous variables are described by their mean and standard deviation

Statistic differences between after the first and the second lockdown was tested using the Mann-Whitney-U test

## Discussion

Since the World Health Organization (WHO) classified COVID-19 as a pandemic, SARS-CoV-2 has impacted life around the world [28]. Without sufficient medication and adequate coverage rates of vaccination, preventative measures have been and still are the only way to contain the COVID-19 pandemic [29]. As part of these precautions, the WHO and the German Federal Ministry of Health recommend hygiene measures such as hand disinfection [30, 31]. To implement these measures, the campaign “AHA” (“Abstand, Hygiene, Alltagsmaske” – “Distance, Hygiene, Facemask”) was launched in Germany [31]. Other preventative measures, such as lockdowns, have been implemented all over the world [5].

Although the world’s population has been threatened by pandemics in every decade of the last 30 years, none had such a strong impact on daily life but also on the hygiene behavior of medical students. For example, during the H1N1 influenza pandemic, neither the awareness of H1N1 influenza increased nor the compliance to hygiene protocols by medical students (hand hygiene, use of mouth and nose protection) [16, 20, 21]. The hygiene behavior of medical students remained unaffected [17]. Since these studies have been based on surveys, a discrepancy between self-perception and hygiene behavior might have been possible. Nonetheless, the literature on the COVID-19 pandemic, also based primarily on self-reported questionnaires, suggests a stronger impact of SARS-CoV-2 on medical students’ hygiene knowledge, behavior and adherence [15, 18, 19, 22–25, 32, 33].

During the H1N1 influenza pandemic, the perceived individual risk of infection appeared to be a strong indicator for the level of pandemic awareness and observance of hygiene behavior among medical students [16]. Due to the more severe course of disease, increased lethality, and wider spread, medical students might perceive the risk of a SARS-CoV-2 infection as higher than they did with the H1N1 influenza [34, 35]. Pandemic containment measures, such as mandatory face masks, also increased the perceived presence of COVID-19 in everyday life [36, 37]. Moreover, the media landscape has changed since the H1N1 influenza pandemic, making information widely and easily accessible. While medical students received information about the H1N1 influenza pandemic through newspapers, medical journals or television, current medical students are more likely to obtain information about SARS-CoV-2 through social media [18, 20, 23, 32, 38, 39]. It can be assumed that social media facilitates medical students self-reported high levels of awareness and knowledge about SARS-CoV-2 as well as compliance to pandemic containment measures and hygiene standards, as indicated in several studies [15, 18, 19, 22–25, 32, 33]. The increase in hygiene compliance demonstrated in this study corresponds to the self-reported high level of knowledge about SARS-CoV-2 and the compliance regarding its containment. In contrast to these results, two questionnaire-based studies describe high levels of awareness and knowledge about SARS-CoV-2, also reported insufficient implementation of hygiene measures among medical students in Mumbai and Egypt [8, 38]. This apparent discrepancy might be explained by the different pandemic stages, during which these studies were conducted. Since the studies were conducted shortly after the pandemic was declared, the examined medical students might have been less familiar with the pandemic and its preventative measures. Furthermore, 80% of accumulated COVID-19 cases and death were reported in Europe and America at the time of this study [40]. The German medical students could have perceived the risk of contracting SARS-CoV-2 as higher compared to the previously studied participants in India or Egypt, driving the conflicting results. Apart from this, a multitude of other factors could potentially have influenced the outcomes of the studies. Further research is needed to confirm, whether these findings can be transferred to other American or European states. This study’s results regarding the hand hygiene compliance before and after the second lockdown further substantiate the hypothesis of a relationship between risk perception and hygiene compliance. Since more medical students properly implemented hand disinfection after the second lockdown as compared to the first, it seems plausible that a prolonged exposure to pandemic containment measures led to increased awareness, which in turn resulted in higher rates of hand disinfection after medical glove removal.

Overall, however, rates of hand disinfection after venipuncture were inadequate in all studied cohorts. This may be attributed to the utilization of noninfectious simulation manikins in this study. Hence, hand disinfection after venipuncture in this study served only a minor role in self-protection and self-cleaning, which serve as the main motivating factors for medical students’ hand disinfection after patient contact [41].

Moreover, it remains unclear if and how how the hygiene behavior of medical students exposed to COVID-19 changes after the pandemic or the course of their studies. In the literature, medical students with more experience are associated with higher awareness and compliance to SARS-CoV-2 containment measures [27, 38]. This could be due to a greater amount of medical background knowledge, simplifying the understanding of COVID-19 relevant information. Nonetheless, the literature also suggests, that the hygiene behavior of medical students without the influence of a pandemic decreases during their medical training [41–44]. Whether and how these two effects may influence each other not only during, but also after the COVID-19 pandemic needs to be investigated further.

Surprisingly, the pandemic-related improvement in hygiene compliance was not reflected in the overall venipuncture score, as medical students performed worse in terms of work structure, successful venipuncture, and patient education during the COVID-19 pandemic. The decreased doctor-patient communication could be a result of pandemic-induced psychological distress or stress due to a lack of hands-on practice opportunities since stress reportedly correlates with decreased doctor-patient communication [9, 29, 45–49]. Additionally, pandemic containment measures and the switch to online teaching at universities may have caused to a lack of hands-on practice opportunities for medical students [45, 50]. Consequently, medical students were unable to become sufficiently familiarized with a proper structure for practical work and to practice complex procedures, such as venipuncture. Since medical students performed even worse after the second than after the first lockdown, the accumulation of such missed practice opportunities might further affect the quality of medical students’ practical skills.

Even though the pandemic positively impacted the hygiene behavior of medical students, their impaired practical skills must be addressed to restore the former standard of practical medical education and ensure patients’ well-being.

## Conclusion

This study found an overall increase in compliance with hygiene measures by medical students during the COVID-19 pandemic. Hand disinfection after patient contact was performed more frequently during the second lockdown as opposed to the first lockdown. It can be assumed that the COVID-19 pandemic and its containment measures increased medical students’ awareness of hygiene. However, it remains to be seen whether the hygiene compliance of medical students will persist after the pandemic. Regardless of this, measures should be taken to reinforce this behavior and pass it on to future generations of prospective physicians.

The observed shortcomings of medical students during the pandemic in terms of structured work, doctor-patient communication, and venipuncture should be further investigated to identify possible strategies to compensate for these deficiencies going forward.

### Limitations

Since this unicentric study was only conducted with medical students at the University of Cologne, a sampling bias cannot be ruled out. Thus, the results cannot be generalized to medical students from other locations.

Furthermore, this study was realized in an examination setting, so the collected data may deviate from the behavior in the clinical setting. Also, medical students’ hygiene behaviors and practical skills are a dynamic process, so a single observation time points might not capture these skills accurately.

It must also be assumed that the teachers as well as the data collectors could not escape the effects of the COVID-19 pandemic. Sensitization of the data collectors to hygiene during the COVID-19 pandemic, and thus an exaggerated assessment of medical students’ hygiene compliance by the same, thus cannot be ruled out. Another risk of a case control study could be that confounding factors might not have been identified, which in turn could have led to confounding bias.

At the same time, the retrospective nature of this study only allows conclusions about the correlation of hygiene, practical skills, and the COVID-19 pandemic. Thus, possible causalities need to be investigated by further prospective studies.

## Supplementary Information

**Additional file 1: Appendix 1.** Rating of the 11-item questionnaire.

**Additional file 2: Appendix 2.** Test of normal distribution prior and after the first COVID-19 lockdown.

**Additional file 3: Appendix 3.** Test of normal distribution for the different student cohorts.

## Data Availability

The dataset generated and analyzed during this study are available from corresponding author on reasonable request.

## Abbreviations

OSCE: Objective structured clinical examinations
SARS-CoV-2: Severe acute respiratory syndrome coronavirus type 2
COVID-19: Coronavirus disease 2019
