# Supplementary material for: The impact of COVID-19 on medical students’ practical skills and hygiene behavior regarding venipuncture: a case control study
Source: BMC Med Educ. 2022 Jul 19;22:558. doi: 10.1186/s12909-022-03601-6 (PMC9294821; doi:10.1186/s12909-022-03601-6)
Supplement: Supplementary file 1 — Additional file 1: Appendix 1. Rating of the 11-item questionnaire. [file 12909_2022_3601_MOESM1_ESM.docx]

| **Appendix 1: Rating of the 11-item questionnaire** | | | |
| --- | --- | --- | --- |
| **Item** | **0 Point** | **¼-½ Point** | **½-1 Point** |
| **Informing the patient about the procedure** | The patient was not informed about the procedure. |  | The patient was informed about the procedure. |
| **Preparation of the needed material** | The Sharp-safe or more than a single needed material is missing. | A single needed material is missing. | All needed material is prepared. |
| **Hand disinfection prior to patient contact** | Hands are not disinfected prior to patient contact nor medical gloves are used. | Medical gloves are worn without prior hand disinfection. | Hands are disinfected prior to patient contact. |
| **Application and deposition of the tourniquet** | The tourniquet is not applied or disposed of correctly. | The tourniquet is applied correctly but deposited incorrectly. | The tourniquet is correctly applied and deposited. |
| **Disinfection of the puncture site** | The puncture site is not disinfected, or it is palpated after the disinfection. |  | The puncture site is disinfected correctly. |
| **30 second application time for the disinfectant** | 30 seconds exposure time of disinfectant is not considered. |  | 30 seconds exposure time of disinfectant is considered. |
| **Needle safety while puncturing the vein** | Needle is contaminated or bent by the student. |  | Needle is not contaminated nor bent by the student. |
| **Venipuncture** | The vein was not successfully punctured. | The vein was only successfully punctured the second time. | The vein was successfully punctured. |
| **Discarding of the puncture needle** | The puncture needle is not discarded into the Sharp-safe. | The puncture needle is not directly discarded into the Sharp-safe. | The puncture needle is directly and correctly discarded into the Sharp-safe. |
| **Hand disinfection after patient contact** | Hands are not disinfected after discarding the medical gloves. |  | Hands are disinfected after discarding the medical gloves. |
| **Structured work** | The work is not structured. | The work is partly structured. | The work is structured. |
