# Supplementary material for: The impact of COVID-19 on medical students’ practical skills and hygiene behavior regarding venipuncture: a case control study
Source: BMC Med Educ. 2022 Jul 19;22:558. doi: 10.1186/s12909-022-03601-6 (PMC9294821; doi:10.1186/s12909-022-03601-6)
Supplement: Supplementary file 2 — Additional file 2: Appendix 2. Test of normal distribution prior and after the first COVID-19 lockdown. [file 12909_2022_3601_MOESM2_ESM.docx]

| **Appendix 2: Test of normal distribution prior and after the first Covid-19 lockdown.** | | | | |  |
| --- | --- | --- | --- | --- | --- |
| Kolmogorov-Smirnova | | Statistic | df | P-value |  |
|  |  |  |  |  |  |
| Relative score in hygiene related tasks | Before the first lockdown | 0.063 | 355 | 0.002 |  |
|  | After the first lockdown | 0.095 | 371 | 0.000 |  |
| Relative score in OSCE 1 | Before the first lockdown | 0.081 | 355 | 0.000 |  |
|  | After the first lockdown | 0.087 | 371 | 0.000 |  |
| Complete preparation of the materials for the PIV | Before the first lockdown | 0.314 | 355 | 0.000 |  |
|  | After the first lockdown | 0.349 | 371 | 0.000 |  |
| Hand disinfection prior to patient contact and PIV placement | Before the first lockdown | 0.497 | 355 | 0.000 |  |
|  | After the first lockdown | 0.536 | 371 | 0.000 |  |
| Tourniquet usage | Before the first lockdown | 0.325 | 355 | 0.000 |  |
|  | After the first lockdown | 0.410 | 371 | 0.000 |  |
| Disinfection of the PIV puncture site | Before the first lockdown | 0.506 | 355 | 0.000 |  |
|  | After the first lockdown | 0.537 | 371 | 0.000 |  |
| 30 second application time for the disinfectant considered | Before the first lockdown | 0.541 | 355 | 0.000 |  |
|  | After the first lockdown | 0.538 | 371 | 0.000 |  |
| Venipuncture during PIV placement | Before the first lockdown | 0.302 | 355 | 0.000 |  |
|  | After the first lockdown | 0.246 | 371 | 0.000 |  |
| Sterile venipuncture needle | Before the first lockdown | 0.514 | 355 | 0.000 |  |
|  | After the first lockdown | 0.508 | 371 | 0.000 |  |
| Discarding of the PIV puncture needle | Before the first lockdown | 0.319 | 355 | 0.000 |  |
|  | After the first lockdown | 0.296 | 371 | 0.000 |  |
| Informing the patient | Before the first lockdown | 0.516 | 355 | 0.000 |  |
|  | After the first lockdown | 0.464 | 371 | 0.000 |  |
| Structured work process | Before the first lockdown | 0.454 | 355 | 0.000 |  |
|  | After the first lockdown | 0.414 | 371 | 0.000 |  |
| Hand disinfection after patient contact | Before the first lockdown | 0.494 | 355 | 0.000 |  |
|  | After the first lockdown | 0.378 | 371 | 0.000 |  |
| a. Correction for significance according to Lilliefors. | | | | |  |
