# Supplementary material for: The impact of COVID-19 on medical students’ practical skills and hygiene behavior regarding venipuncture: a case control study
Source: BMC Med Educ. 2022 Jul 19;22:558. doi: 10.1186/s12909-022-03601-6 (PMC9294821; doi:10.1186/s12909-022-03601-6)
Supplement: Supplementary file 3 — Additional file 3: Appendix 3. Test of normal distribution for the different student cohorts. [file 12909_2022_3601_MOESM3_ESM.docx]

| **Appendix 3: Test of normal distribution for the different student cohorts.** | | | | |  |
| --- | --- | --- | --- | --- | --- |
| Kolmogorov-Smirnova | | Statistic | df | P-value |  |
|  |  |  |  |  |  |
| Relative score in OSCE | Winter semester 2018/2019 | 0.100 | 191 | 0.000 |  |
|  | Summer semester 2019 | 0.079 | 164 | 0.015 |  |
|  | Summer semester 2020 | 0.096 | 180 | 0.000 |  |
|  | Winter semester 2020/2021 | 0.072 | 191 | 0.018 |  |
| Complete preparation of the materials. | Winter semester 2018/2019 | 0.319 | 191 | 0.000 |  |
|  | Summer semester 2019 | 0.314 | 164 | 0.000 |  |
|  | Summer semester 2020 | 0.298 | 180 | 0.000 |  |
|  | Winter semester 2020/2021 | 0.397 | 191 | 0.000 |  |
| Hand disinfection prior to patient contact. | Winter semester 2018/2019 | 0.535 | 191 | 0.000 |  |
|  | Summer semester 2019 | 0.434 | 164 | 0.000 |  |
|  | Summer semester 2020 | 0.536 | 180 | 0.000 |  |
|  | Winter semester 2020/2021 | 0.536 | 191 | 0.000 |  |
| Tourniquet usage. | Winter semester 2018/2019 | 0.354 | 191 | 0.000 |  |
|  | Summer semester 2019 | 0.291 | 164 | 0.000 |  |
|  | Summer semester 2020 | 0.423 | 180 | 0.000 |  |
|  | Winter semester 2020/2021 | 0.398 | 191 | 0.000 |  |
| Disinfection of the PIV puncture site. | Winter semester 2018/2019 | 0.496 | 191 | 0.000 |  |
|  | Summer semester 2019 | 0.516 | 164 | 0.000 |  |
|  | Summer semester 2020 | 0.538 | 180 | 0.000 |  |
|  | Winter semester 2020/2021 | 0.536 | 191 | 0.000 |  |
| 30 second application time for the disinfectant considered. | Winter semester 2018/2019 | 0.537 | 191 | 0.000 |  |
|  | Summer semester 2019 | 0.538 | 164 | 0.000 |  |
|  | Summer semester 2020 | 0.540 | 180 | 0.000 |  |
|  | Winter semester 2020/2021 | 0.534 | 191 | 0.000 |  |
| Venipuncture during PIV placement | Winter semester 2018/2019 | 0.333 | 191 | 0.000 |  |
|  | Summer semester 2019 | 0.269 | 164 | 0.000 |  |
|  | Summer semester 2020 | 0.304 | 180 | 0.000 |  |
|  | Winter semester 2020/2021 | 0.206 | 191 | 0.000 |  |
| Sterile venipuncture needle | Winter semester 2018/2019 | 0.516 | 191 | 0.000 |  |
|  | Summer semester 2019 | 0.511 | 164 | 0.000 |  |
|  | Summer semester 2020 | 0.510 | 180 | 0.000 |  |
|  | Winter semester 2020/2021 | 0.505 | 191 | 0.000 |  |
| Discarding of the PIV puncture needle | Winter semester 2018/2019 | 0.306 | 191 | 0.000 |  |
|  | Summer semester 2019 | 0.334 | 164 | 0.000 |  |
|  | Summer semester 2020 | 0.270 | 180 | 0.000 |  |
|  | Winter semester 2020/2021 | 0.324 | 191 | 0.000 |  |
| Informing the patient | Winter semester 2018/2019 | 0.525 | 191 | 0.000 |  |
|  | Summer semester 2019 | 0.504 | 164 | 0.000 |  |
|  | Summer semester 2020 | 0.483 | 180 | 0.000 |  |
|  | Winter semester 2020/2021 | 0.444 | 191 | 0.000 |  |
| Structured work process | Winter semester 2018/2019 | 0.497 | 191 | 0.000 |  |
|  | Summer semester 2019 | 0.399 | 164 | 0.000 |  |
|  | Summer semester 2020 | 0.466 | 180 | 0.000 |  |
|  | Winter semester 2020/2021 | 0.364 | 191 | 0.000 |  |
| Hand disinfection after patient contact | Winter semester 2018/2019 | 0.462 | 191 | 0.000 |  |
|  | Summer semester 2019 | 0.525 | 164 | 0.000 |  |
|  | Summer semester 2020 | 0.352 | 180 | 0.000 |  |
|  | Winter semester 2020/2021 | 0.423 | 191 | 0.000 |  |
| a. Correction for significance according to Lilliefors. | | | | |  |
